# Evaluating Therapeutic Efficacy of Intravesical Xenogeneic Urothelial Cell Treatment Alone and in Combination with Chemotherapy or Immune Checkpoint Inhibition in a Mouse Non-Muscle-Invasive Bladder Cancer Model

**DOI:** 10.3390/cancers17152448

**Published:** 2025-07-24

**Authors:** Chih-Rong Shyr, Ching-Feng Wu, Kai-Cheng Yang, Wen-Lung Ma, Chi-Ping Huang

**Affiliations:** 1 Department of Medical Laboratory Science and Biotechnology, China Medical University, Taichung 404, Taiwan; 2 eXCELL Biotherapeutics Inc., Taichung 404, Taiwan; 3 Department of Medicine, Urology Division, China Medical University and Hospital, Taichung 404, Taiwan; johnucmecena@gmail.com (C.-F.W.);; 4 Graduate Institute of Biomedical Sciences, Ph.D. Program for Health Science and Industry, School of Medicine, China Medical University, Taichung 404, Taiwan; maverick@mail.cmu.edu.tw

**Keywords:** non-muscle-invasive bladder cancer, xenogeneic urothelial cell, intravesical, xenoantigen, rejection, antitumor immunity

## Abstract

**Background/Objectives**: Bladder cancer is a malignant disease that causes more than 199,922 deaths a year globally, in which ~75% of all newly diagnosed cases are non-muscle-invasive bladder cancer (NMIBC). Despite a number of treatments available, most NMIBC patients with high-grade tumors eventually recur. To add a novel therapy to complement the deficits of the current treatments, this study assesses the antitumor activity and mechanisms of action of intravesical xenogeneic urothelial cell (XUC) treatment as monotherapy and in combination with either chemotherapy or immune checkpoint inhibition (ICI). **Methods**: The orthotopic NMIBC graft tumor-bearing mice were randomly assigned into different treatment groups, receiving either intravesical XUCs, gemcitabine, anti-programmed death-ligand 1 (PD-L1) antibodies alone or in combination with gemcitabine or anti-PD-1 antibodies. The tumor responses, survival, and immune reactions were analyzed. **Results**: Intravesical XUC treatment exhibited significantly more antitumor activity to delay tumor progression than the control group and a similar effect to chemotherapy and ICI. In addition, there were significantly higher effects in the combined groups than single treatments. Immune tumor microenvironment and immune cell proliferation, cytotoxicity, and cytokine secretion were also activated by XUC treatment. Moreover, the combined groups have the highest effects. **Conclusions**: In vivo and ex vivo studies showed increased antitumor efficacy and immune responses by intravesical XUC treatment in single and combined treatments, suggesting a potential utility of this xenogeneic cell immunotherapeutic agent. Intravesical XUC treatment has the potential to address the substantial unmet need in NMIBC therapy as a bladder-sparing treatment option for NMIBC.

## Simple Summary

The high recurrence rate and potential progression to muscle-invasive and metastatic stages have been great challenges for the management of high-risk non-muscle-invasive bladder cancer despite a variety of treatment options being available, including surgery, chemotherapy, and immunotherapy. To test a new immunotherapeutic approach to complement the current therapeutic modalities to achieve durable responses, we investigated the antitumor effect of a novel intravesical xenogeneic urothelial cell treatment in a murine non-muscle-invasive bladder cancer (NMIBC) model. Our study aimed to assess the potential use of this innovative therapeutic modality as a treatment option for NMIBC.

## 1. Introduction

Bladder cancer (BC) is a major cause of morbidity and mortality worldwide, with an estimated 549,393 cases, causing an estimated 199,922 deaths in a year [1]. Approximately 75% of the patients are diagnosed with non-muscle-invasive bladder cancer (NMIBC), which has a high disease recurrence rate of 50%–70% and 10%–15% of patients progress to muscle-invasive bladder cancer (MIBC) and metastatic disease in the current standard of care treatment [2,3]. The standard of care for patients with high-grade (HG), intermediate, and high-risk NMIBC is complete transurethral resection of the tumor followed by induction and maintenance immunotherapy with intravesical Bacillus Calmette–Guérin (BCG) or intravesical chemotherapy [4]. Intravesical BCG immunotherapy using a live attenuated form of mycobacterium bovis has been a standard therapeutic approach for decades, and yet eventually fails in up to 50% of patients, and approximately 50% of those failures occur within the first 6 months [5]. While current guidelines advise offering early radical cystectomy as an option for patients who have high-risk disease after BCG failure, radical cystectomy is associated with considerable morbidity and mortality [6,7]. Furthermore, a number of patients are unfit for cystectomy or prefer to preserve bladder function due to the risks of co-morbid conditions and short and long-term complications, which impair quality of life [8].

For BCG unresponsive NMIBC, intravesical valrubicin was the first treatment approved by the United States Food and Drug Administration (FDA) when cystectomy is not eligible due to morbidity and mortality concerns [9]. In addition, systemic pembrolizumab, a PD-1 immune checkpoint inhibitor, was approved in this setting for patients who declined or were ineligible for radical cystectomy [10]. Intravesical nadofaragene firadenovec therapy was also approved for BCG unresponsive NMIBC, which is a gene therapy using the interferon alfa-2b gene expressed adenovirus [11]. Intravesical nogapendekin alfa inbakicept (NAI), an IL-15RαFc superagonist, plus BCG was the most recently approved treatment for patients with BCG-unresponsive NMIBC. An alternative off-label therapeutic option is intravesical gemcitabine/docetaxel therapy that sequences gemcitabine instillation followed by docetaxel [12].

Despite a variety of treatments available for BCG-unresponsive NMIBC, most patients eventually lose the responses. For the treatment of intravesical NAI plus BCG, which has been demonstrated as the most effective approved treatment so far, the patients with a duration of response ≥18 months were 24% [13]. Thus, more effective and bladder-sparing options are needed to broaden the landscape of treatment options beyond BCG, chemotherapy, anti-PD1 antibody, cytokine, and viral gene therapy to improve the quality and quantity of bladder cancer patients’ lives.

Xenogeneic cells of different tissue types are proposed to be used as an immunotherapeutic agent for cancer immunotherapy by stimulating the body’s immune system with rejection immune responses that boost antitumor immunity [14]. Comparing the molecular and cellular mechanisms of xenogeneic cell rejection immune responses and antitumor immune reactions by the host body’s immune systems, there are many similarities between xenoantigens and tumor-specific antigens to elicit innate and adaptive immunity [14,15,16]. The mechanisms of action in xenogeneic cell rejection immune responses and cancer immunity cycle are very similar, both involving the activation of innate and adaptive immunity [15,17], thus enabling xenogeneic cells as a cellular product for treating cancers. The intravesical xenogeneic urothelial therapy has previously demonstrated the ability to inhibit tumor progression in orthotopic mouse bladder cancer models through activating antitumor immune responses [18]. Furthermore, intralesional/intratumoral injection of xenogeneic urothelial cells alone suppressed tumor growth and the efficacy was enhanced when combining chemotherapy on both injected and noninjected tumors in heterotopic graft bladder tumor models [19]. Intratumoral immune cell infiltration and systemic activation of immune cell cytotoxic activity, cytokine IFNγ production, and proliferation ability as well as elevated chemokine CXCL9/10/11 levels in tumors were observed in mice receiving intralesional/intratumoral therapy [19]. These data suggest that xenogeneic urothelial cells could be used as a promising immunotherapeutic agent in the treatment of bladder cancer as a local therapy.

In the present study, we investigated antitumor activities of intravesical xenogeneic urothelial cells as monotherapy or in combination with intravesical chemotherapy or immune checkpoint inhibition using an orthotopic NMIBC bladder tumor mouse model. Our results revealed increased activities in suppressing tumor progression, altering tumor immune microenvironment, and enhancing immune cell proliferation and cellular functions reacted to xenogeneic and cancer cells with intravesical XUC treatment alone. The induced immune rejection of bladder tumors was further increased in combined treatments.

## 2. Materials and Methods

### 2.1. Cell

The MB49 cell line, derived from the bladder epithelial cells of C57BL/Icrf-a mice [20], was cultivated in complete DMEM (Gibco, 12100061, Carlsbad, CA, USA) supplemented with 10% (*v*/*v*) fetal bovine serum (FBS; Gibco, A31605, Carlsbad, CA, USA) and 100 U/mL penicillin–streptomycin (P/S; Gibco, 10378016, Carlsbad, CA, USA). Cells were incubated at 37 °C in a humidified incubator containing 5% (*v*/*v*) CO_2_. Xenogeneic urothelial cells (XUC) were isolated from bladders of specific pathogen-free (SPF) pigs obtained from the Agricultural Technology Research Institute, Taiwan, following previously reported protocols for porcine urothelial cell culture [18]. Only cells at passages 2–10 were utilized in experiments, and all cells were tested negative for mycoplasma, bacteria, yeast, and fungi prior to use.

### 2.2. Mice

Six- to ten-week-old female C57BL/6 mice were obtained from the National Laboratory Animal Center. All mouse experiments and procedures were approved by the Animal Center of China Medical University Hospital (Protocol No: CMUIACUC-2021-089). The orthotopic MB49-luc non-muscle-invasive bladder tumor murine model was established with the following procedures. A total of 2 × 10^5^ MB49-luc tumor cells suspended in 100 μL of 1× phosphate-buffered saline (PBS) were instilled into mouse bladders after being pre-treated with 100 μL of poly-L-lysine solution (0.1 mg/mL) (Merck, A-005-C, Rahway, NJ, USA) for 30 min. The cells were allowed to dwell in the bladders for 50–60 min. Then, 24-gauge catheters were utilized for all intravesical administrations for tumor implantation and drug treatments. Tumor growth was monitored by bioluminescence imaging (BLI) every 7 days post-implantation using the Xenogen IVIS 200 (Xenogen, Alameda, CA, USA). Mice were injected intraperitoneally (I.P.) with 4 mg/mouse of D-luciferin (PerkinElmer, 122799, Shelton, CT, USA) and the BLI was captured after 10 min.

For XUC treatment, cryopreserved 1 × 10^6^ XUC cells were thawed by incubating in a 37 °C water bath and were intravesically instilled into the mice’s bladder and allowed to dwell for 1 h. In the combination treatment with intravesical gemcitabine, a total of 0.5 mg gemcitabine was instilled into the mice bladders via intravesical administration. The drug remained in the bladder for 20 min, followed by a 10 min saline wash. After washing, the mice were treated with XUC for 1 h. This treatment was performed weekly, with the mice under anesthesia. For anti-PD1 antibody (Ichorbio, ICH1132, Wantage, Oxfordshire, UK) treatment, I.P. administration (200 µg/treatment) was performed.

Each treatment group originally included 5 mice, but if the mice died early or met the humane endpoint, more mice were added. Animals were monitored daily, and humane endpoint criteria were followed, including body weight loss >20%, impaired mobility, hematuria, or tumor ulceration. Animals that met these criteria were euthanized before day 28, accounting for discrepancies between survival curves and endpoint bladder weight quantifications.

### 2.3. Immunohistochemistry (IHC)

Bladders harvested from mice in each treatment group were fixed in 4% paraformaldehyde buffer. Tissue sections were stained with antibodies against Ki67 (Abcam, ab15580, Cambridge, Cambridgeshire, UK), CD4 (GeneTex, GTX85525, Irvine, CA, USA), CD8 (GeneTex, GTX53126, Irvine, CA, USA), CD56 (Sino Biological, 108577-T08, Beijing, China), CD68 (Abcam, ab125212, Cambridge, Cambridgeshire, UK), and CD11b (Abcam, ab9535, Cambridge, Cambridgeshire, UK) following the manufacturer’s standard protocols using an automated Leica Bond III-autostainer (Leica Biosystems, Nußloch, Baden-Württemberg, Germany). Positive cells were quantified by counting three to four random high-power fields (HPF) at 400× magnification, and the results were averaged as the number of positive cells per field.

### 2.4. Terminal Deoxynucleotidyl Transferase (TdT)-Mediated dUTP Nick End Labeling (TUNEL) Assay

DNA fragmentation in apoptotic cells within tumor sections was detected by the TUNEL BrightGreen Apoptosis Detection Kit (ACE Biolabs, CE1007, Taoyuan, Taiwan), according to the manufacturer’s protocol. Briefly, paraffin-embedded tumor sections were deparaffinized, followed by incubation in TdT incubation buffer and DPI solution. Fluorescent images were captured using a Nikon Eclipse 80i fluorescence microscope (Nikon, Tokyo, Japan) equipped with a CCD camera. Fluorescently labeled apoptotic cells were counted, and the percentage of positive cells was calculated.

### 2.5. Immune Cell Proliferation Assay

Carboxyfluorescein diacetate succinimidyl ester (CFDA-SE) (Invitrogen, C1157, Carlsbad, CA, USA) was utilized to monitor cell proliferation. Immune cells were isolated from the spleens of tumor-bearing mice subjected to different treatments and incubated for 20 min in the dark with 5 μM CFDA-SE in 1xPBS. The assay was conducted by pre-seeding 1 × 10^4^ target xenogeneic cells or MB49 tumor cells. Once target cells adhered, they were co-cultured with 5 × 10^4^ CFDA-SE-labeled effector immune cells from the spleens (E/T ratio 5:1) for 3 days. Cells were collected by centrifugation at 3000 rpm and resuspended in 1xPBS. The intensity of CFDA-SE fluorescence in lymphocytes was measured using a FACSCalibur flow cytometer (BD Biosciences, Franklin Lakes, NJ, USA) and analyzed with FlowJo Software (version 10).

### 2.6. Cytokine Enzyme-Linked Immunosorbent Assay (ELISA)

The supernatant from the co-culture assay was collected to determine cytokine secretion. The levels of IFNγ were quantified using ELISA assay kits (Biolegend, 430815, San Diego, CA, USA), following the manufacturer’s instructions. The absorbance values were measured using a Multiskan GO microplate reader (ThermoFisher, Waltham, MA, USA).

### 2.7. Immune Cell Cytotoxic Activity

CFDA-SE-labeled XUCs and MB49 tumor cells were used as target cells to assess the cytotoxic activity of effector splenic lymphocytes. Target cells were pre-cultured in a monolayer for 24 h, followed by co-culturing with effector lymphocytes isolated from the spleens of mice subjected to different treatments at an effector/target (E:T) ratio of 10:1. After 16–18 h of incubation, effector cells were removed, and the fluorescence intensity of the remaining adherent target cells was measured by a SpectraMax iD3 plate reader (Molecular Devices, San Jose, CA, USA). The fluorescence intensity of CFDA-SE-labeled target cells without co-culture with effector cells served as the baseline. The relative cytotoxic activity of effector lymphocytes from different treatment groups was calculated from triplicate samples using the formula [(Baseline intensity − experimental intensity)/(Baseline intensity)] and expressed as a percentage.

### 2.8. Quantification of Immune Effector-Target Cell Conjugate Formation by Using Imaging Flow Cytometry

To analyze cell conjugate formation, conjugates were formed between splenocytes and MB49 cells. Briefly, 2 × 10^6^ MB49 cells were centrifuged and immediately resuspended in 100 μL of RPMI 1640 medium (Gibco, 31800022, Carlsbad, CA, USA) containing 2 × 10^7^ immune cells isolated from the spleens of treated mice. The cell mixture was incubated at 37 °C for 45 min, followed by gentle vortexing (10 s at 1000 rpm), during which 100 μL of paraformaldehyde (final concentration 2%) was added to fix the cells for 10 min at room temperature. Paraformaldehyde was removed by washing the cells with washing buffer (PBS containing 1% BSA), after which the cells were permeabilized by incubating in the same buffer with an additional 0.1% Triton X-100 for 15 min at room temperature. The cells were then stained with fluorophore-labeled antibodies or compounds: CD3-AF647 (1:25) (BioLegend, 301620, San Diego, CA, USA), Alexa Fluor 488 Phalloidin (1:5000) (Invitrogen, A12379, Carlsbad, CA, USA), and DAPI (1:3000) (Invitrogen, D1306, Carlsbad, CA, USA). The staining was carried out by incubating the cells at room temperature in the dark for 1 h. Cells were then washed three times with washing buffer, centrifuged at 300× *g* for 10 min at room temperature, and resuspended in 100 μL of PBS for imaging flow cytometry.

To identify the contact zones between CD3-positive lymphocytes and MB49 cells, image acquisition and data analysis were performed using imaging flow cytometry software, including ImageStream (IS100) INSPIRE (version 201.1.0.765)and IDEAS (version 6.2). Total events were gated based on true T-cell/MB49 conjugates, and a DAPI-dependent valley mask was applied to delineate the interface between these coupled cells. The immune conjugate mask was defined as the combination of the valley mask and a T-cell mask utilizing CD3 staining. The T-cell mask was set at the upper 60% of the CD3 fluorescence signal (Threshold: M11, Ch11, 60), while an additional mask was employed to define the immune conjugate (Valley: M07, Ch07, 3; Threshold: M11, Ch11, 60). Mature immune conjugates were identified by the co-localization and enrichment of F-actin and CD3 within the T-cell/MB49 cell conjugates. This was quantified by calculating the ratio of the mean pixel intensities of CD3 fluorescence within the conjugate region to that in the T-cell mask (Mean Pixel_Conjugate_Ch11/Mean Pixel_M11_Ch11). Additionally, the mean pixel intensities of F-actin within the conjugate were compared to the overall mean intensity of F-actin in the T-cells (Mean Pixel_Conjugate_Ch2/Mean Pixel_M11_Ch2). Conjugates were considered mature when both of these ratios exceeded 1. Eventually, each event gated as a mature immune conjugate was visually confirmed.

## 3. Results

### 3.1. Antitumor Activity of Intravesical Xenogeneic Urothelial Cell (XUC) Treatment as Monotherapy and in Combination with Chemotherapy and Immune Checkpoint Inhibition in the Orthotopic NMIBC Bladder Tumor-Bearing Mice

**Figure 1:**
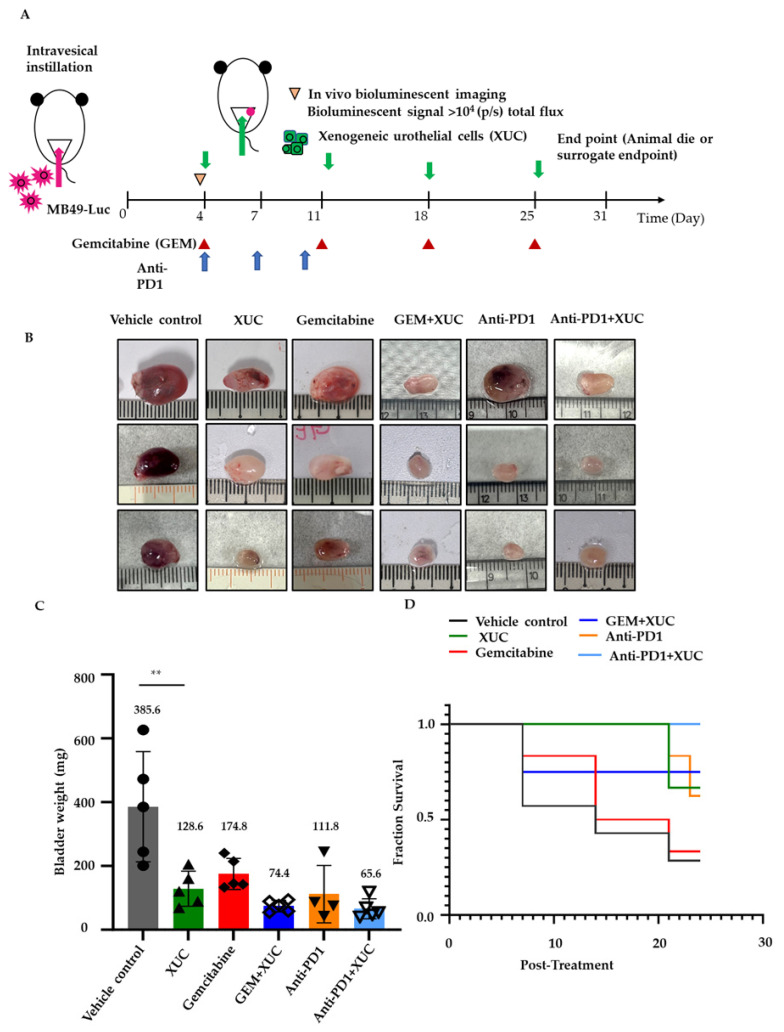
Intravesical xenogeneic urothelial cells (XUC) alone or in combination with intravesical gemcitabine (GEM) or systemic anti-PD1 treatment exhibit in vivo antitumor efficacy in the MB49 syngeneic orthotopic graft NMIBC tumor model. Tumor implantation was performed via intravesical instillation of MB49-luc cells into the bladders of female mice with tumor development monitored by bioluminescence imaging (BLI). When the tumor developed, mice were randomized to one of six treatment groups (vehicle control, XUC, GEM, XUC plus GEM, anti-PD1, or XUC plus anti-PD1). (**A**) Experimental design and treatment schedule. (**B**) Representative gross images of bladders of treated mice. (**C**) Bladder weights of mice from different treated groups were recorded at the end-point of experiments (*n* = 5 for each group). (**D**) The overall survival curves of mice from different treatment groups (vehicle control *n* = 7, XUC *n* = 5, GEM *n* = 7, XUC plus GEM *n* = 5, anti-PD1 *n* = 5, or XUC plus anti-PD1 *n* = 5). A log-rank test verified significant differences among different treatment groups: XUC vs. vehicle control (*p*-value = 0.0889); GEM vs. vehicle control (*p*-value = 0.6942); GEM + XUC vs. vehicle control (*p*-value = 0.0963); anti-PD1 vs. vehicle control (*p*-value = 0.1019); and anti-PD1 + XUC vs. vehicle control (*p*-value = 0.0192). The number on the top of the bar shows the average value. Data are represented as mean ± SD, with significance determined by one-way ANOVA compared to the vehicle control group. ** *p* < 0.005.

We first explored the potential antitumor effects of intravesical administration of XUCs in an orthotopic MB49 NMIBC bladder tumor model. To develop a more effective cancer immunotherapy, we also tested the combination therapy with either chemotherapy to sensitize tumors to the effects of immunotherapy or anti-PD1 immune checkpoint blockade to fully turn on the cancer immunity cycle [21]. We compared the in vivo studies on the therapeutic efficacy of XUC treatment alone or in combination with gemcitabine or anti-PD1 in inhibiting tumor progression. The MB49-Luc orthotopic mouse model, which closely mimics human non-muscle-invasive bladder cancers (NMIBC), was utilized for these experiments. After the tumor developed, mice were treated with either 1 × 10^6^ XUC cells, gemcitabine, anti-PD1 antibodies, or administered alone or in combinations. XUC and gemcitabine (GEM) treatments were intravesically administered weekly for 4 weeks. Intravesical GEM was used since it has been tested as an intravesical agent to treat NMIBC and showed better efficacy and lower toxicity than other chemotherapeutic agents for non-muscle-invasive urothelial cancer [22]. The systemic PD-1 blockade was administered three times intraperitoneally. The study treatment schema is illustrated in Figure 1A. After treatments, XUCs both as a monotherapy and in combination with gemcitabine (GEM) or anti-PD1, significantly inhibited tumor growth (Figure 1B,C). The vehicle control group exhibited an average bladder tumor weight of 385.6 ± 154.5 mg. XUC monotherapy reduced tumor weight to 128.6 ± 49.0 mg, while GEM monotherapy resulted in a tumor weight of 174.8 ± 43.6 mg. The combination of XUC with GEM further enhanced tumor suppression, reducing tumor weight to 74.4 ± 16.0 mg. Similarly, anti-PD1 monotherapy led to a tumor weight of 111.8 ± 78.1 mg, and the combination of XUC with anti-PD1 achieved the most substantial tumor reduction, with an average tumor weight of 65.6 ± 27.9 mg (Figure 1C). In addition to tumor burden reduction, overall survival was significantly prolonged with XUC monotherapy, and was further improved by the combination treatments with GEM or anti-PD1 (Figure 1D).

### 3.2. Intravesical Xenogeneic Urothelial Cell Immunotherapy Treatment Inhibits Tumor Cell Proliferation and Promotes Cell Apoptosis

**Figure 2:**
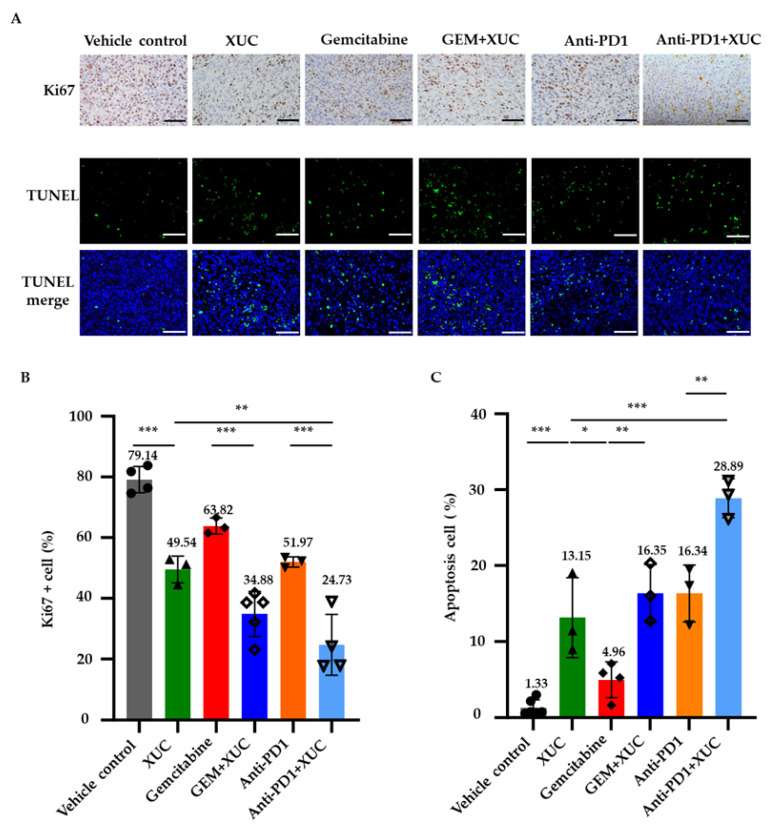
Xenogeneic urothelial cell (XUC) therapy inhibits tumor cell proliferation and induces apoptosis in NMIBC bladder tumors. At the treatment endpoint, bladders from mice of each group were harvested, processed, and sectioned for immunohistochemistry (IHC) analysis of Ki67, a marker of cell proliferation, and TUNEL assay to detect apoptotic cells. Three to four random image fields were captured in each section. (**A**) Representative images of Ki67 staining and TUNEL assay. (**B**) Quantification of Ki67 and (**C**) TUNEL-positive cells was performed on random fields. Scale bars represent 100 μm. The number on the top of the bar shows the average value. Results are expressed as mean ± SD, with statistical significance determined by one-way ANOVA. * *p* < 0.05, ** *p* < 0.005, *** *p* < 0.0005.

To investigate how XUC treatment inhibited tumor progression, we conducted Ki67 immunohistochemistry (IHC) and TUNEL assays to further assess the cellular effects of the antitumor efficacy by XUC treatment on the NMIBC bladder tumors. IHC staining for Ki67 was performed to evaluate tumor cell proliferation, from which the percentage of Ki67-positive cells was calculated relative to the total number of cell nuclei (Figure 2A). On the other hand, the TUNEL assay was used to detect DNA fragmentation, indicative of cell apoptosis. (Figure 2A). In the vehicle control group, 79.14 ± 3.76% of tumor cells were Ki67-positive, indicating a high proliferation rate. XUC monotherapy significantly reduced Ki67 expression to 49.54 ± 3.62%, while GEM monotherapy resulted in a Ki67-positive cell percentage of 63.82 ± 2.12%. The combination of XUC with GEM further suppressed tumor cell proliferation, decreasing Ki67 positivity to 34.88 ± 6.64%. Similarly, anti-PD1 monotherapy reduced the Ki67-positive population to 51.97 ± 1.39%, and the combination of XUC with anti-PD1 resulted in the most pronounced reduction, with only 24.73 ± 8.62% of tumor cells expressing Ki67 (Figure 2B). Conversely, the TUNEL assay revealed an increase in apoptotic cell death following XUC treatment. In the vehicle control group, only 1.33 ± 0.93% of cells were TUNEL-positive. XUC monotherapy increased apoptosis to 13.15 ± 4.3%, whereas GEM alone induced a more modest increase to 4.96 ± 2.03%. The combination of XUC and GEM further enhanced apoptosis, raising TUNEL positivity to 16.35 ± 3.11%. Similarly, anti-PD1 treatment alone led to 16.34 ± 3.06% TUNEL-positive cells, while the combination of XUC with anti-PD1 yielded the highest apoptotic rate, with 28.89 ± 2.09% of tumor cells staining positive (Figure 2C). These findings suggest that XUC treatment exerts a potent antitumor effect by reducing tumor cell proliferation, as evidenced by decreased Ki67 expression, and promoting apoptosis, as shown by increased TUNEL positivity.

### 3.3. Intravesical Xenogeneic Urothelial Cell Treatment Alters the Immune Tumor Microenvironment in NMIBC Bladder Tumors

**Figure 3:**
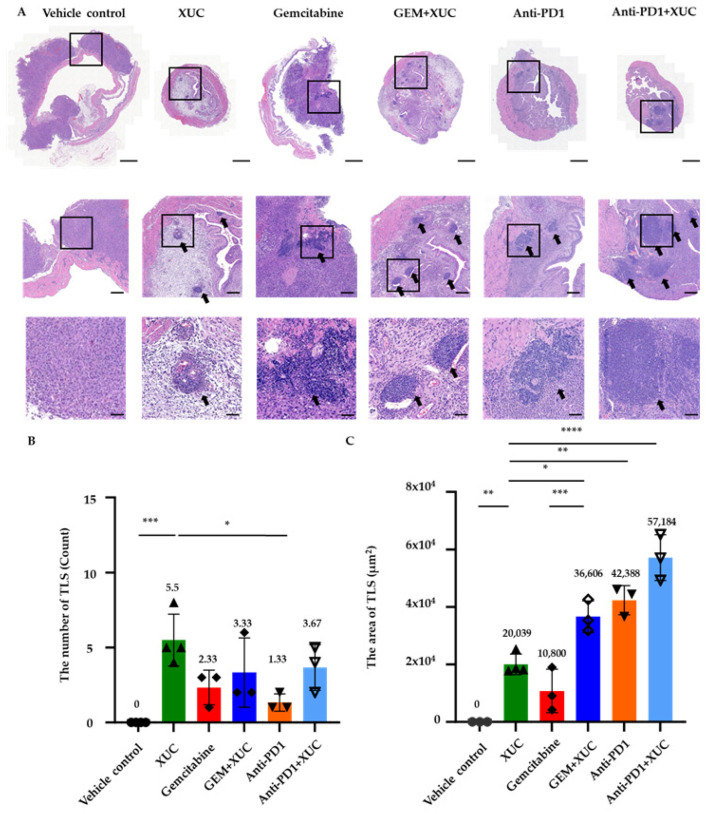
Xenogeneic urothelial cell (XUC) treatment promotes the formation of tertiary lymphoid structures (TLS) in bladder tumor tissues. Bladders from treated mice were sectioned and subjected to hematoxylin and eosin (H&E) staining to evaluate TLS formation. The histological assessment focused on lymphocyte clustering, the presence of germinal center-like structures, and overall tissue architecture to determine TLS development. (**A**) H&E staining of bladder tumor tissues showing representative TLSs located within the bladder sections of MB49 orthotopic tumor-bearing mice. The bottom picture in the lower panel provides a magnified view of the area in the rectangle of the upper panel, with TLSs marked with black arrows. Vehicle control images only show stromal tissue magnification and do not contain TLS. (**B**) The number of TLSs per section and (**C**) the area of TLSs was quantified by ImageJ Software (version 1.54g). Scale bars = 500 μm, 100 μm, and 20 μm, respectively. The number on the top of the bar shows the average value. Results are presented as mean ± SD, with statistical significance determined by one-way ANOVA. * *p* < 0.05, ** *p* < 0.005, *** *p* < 0.001, **** *p* < 0.0001.

To investigate the effects of xenogeneic urothelial cells on the tumor microenvironment, we examined the histological morphology of MB49 bladder tumors with different treatments. Hematoxylin and eosin (H&E) staining of bladder tumor sections from tumor-bearing mice with treatments revealed the presence of immune cell clusters reminiscent of tertiary lymphoid structures (TLS) [23] (Figure 3A). We quantified both the number and area of TLS in the tumor stroma, located beyond the urothelium layer, as an indicator of immune activation in response to tumor implantation (Figure 3B,C). No TLSs were observed in the vehicle control group. In contrast, XUC monotherapy significantly promoted TLS formation, with an average of 5.5 ± 1.5 TLSs per tumor section. GEM alone resulted in a lower TLS count of 2.33 ± 0.94, while the combination of XUC with GEM further increased TLS formation to 3.33 ± 1.89. Similarly, anti-PD1 monotherapy induced 1.33 ± 0.47 TLSs, whereas the combination of anti-PD1 with XUC enhanced TLS formation to 3.67 ± 1.25.

The TLS area followed a similar trend (Figure 3C). In the vehicle control group, no TLS structures were detected. XUC monotherapy induced a significant increase in TLS area, averaging 20,039 ± 3105 μm^2^. GEM treatment alone resulted in a TLS area of 10,800 ± 6154 μm^2^, while the combination of GEM and XUC further expanded the TLS area to 36,606 ± 4496 μm^2^. Anti-PD1 treatment alone led to a TLS area of 42,388 ± 4131 μm^2^, whereas the combination of anti-PD1 and XUC resulted in the most extensive TLS formation, with an average TLS area of 57,184 ± 6473 μm^2^. These results demonstrate that XUC treatment, whether alone or combined with GEM or anti-PD1, promotes the formation of TLS in the tumor microenvironment. Furthermore, the data indicate features reminiscent of secondary lymphoid organs, with clear evidence of germinal centers, particularly in the XUC treatment groups, either alone or in combination with GEM treatment groups. Notably, TLSs were observed both within and adjacent to tumor areas. Due to the full bladder being processed and sectioned for histology rather than dissected tumors only, some tissue sections did not include tumor parts. However, TLSs detected in the bladder stroma still indicate local immune responses induced by tumor presence and therapy.

### 3.4. Intravesical Xenogeneic Urothelial Cell Treatment Promotes Immune Cell Infiltration

**Figure 4:**
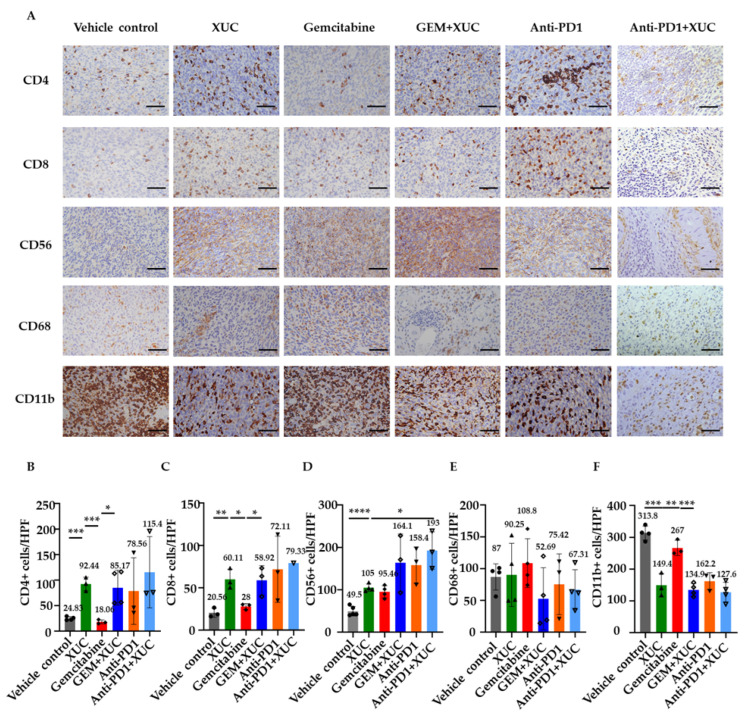
Xenogeneic urothelial cell (XUC) treatment alters immune cell infiltration in tumors. Immunohistochemistry (IHC) staining was performed on bladder tumor tissue sections for immune cell markers: helper T cells (CD4), cytotoxic T cells (CD8), natural killer cells (CD56), monocytes (CD68), and myeloid-derived suppressor cells (CD11b). (**A**) Representative images of bladder tumor tissue sections stained by each antibody. (**B**–**F**) Quantification of immune cell populations in each bladder section was performed by counting cells in three to four random fields per section. The scale bar represents 100 μm. The number on the top of the bar shows the average value. Data are presented as mean ± SD. Statistical significance was determined using multiple *t*-tests. * *p* < 0.05, ** *p* < 0.01, *** *p* < 0.001, **** *p* < 0.0001.

Using immunohistochemical analysis to determine intratumoral immune cell composition, we further counted the numbers of CD4+ and CD8+ T lymphocytes, CD56+ NK cells, and CD68+ monocytes, as well as CD11b+ myeloid-derived suppressor cells (MDSCs) inside the tumors (Figure 4A). T-cell infiltration was observed in all treatments involving XUCs. Quantitative analysis demonstrated that treatments incorporating XUC significantly increased tumor-infiltrating immune cells compared to the vehicle control group, indicating enhanced immune activation. Specifically, the CD4^+^ T cell (Figure 4B) infiltration number increased from 24.83 ± 3.08 in the vehicle group to 92.44 ± 10.9 with XUC monotherapy. The GEM monotherapy group showed no significant increase (18.06 ± 3.28), while combination treatments led to further enhancements, with GEM + XUC at 85.17 ± 29.53, anti-PD1 at 78.56 ± 53.27, and anti-PD1 + XUC at the highest level of 115.4 ± 56.97 per field (Figure 4B). Similarly, the CD8^+^ T cell (Figure 4C) infiltration number rose from 20.56 ± 4.42 in the vehicle group to 60.11 ± 9.66 with XUC monotherapy. GEM alone resulted in a moderate increase to 28.0 ± 2.84. The combination of GEM + XUC led to 58.92 ± 14.16 CD8^+^ T cells, while anti-PD1 monotherapy increased it to 72.11 ± 31.96, with anti-PD1 + XUC reaching the highest at 79.33. Compared to the vehicle control (49.5 ± 10.08), the infiltrating NK cell number rose to 105 ± 7.61 with XUC alone, 95.46 ± 12.33 with GEM monotherapy, 164.1 ± 55.35 with GEM + XUC, 158.4 ± 35.06 with anti-PD1, and peaked at 193 ± 36.16 in the anti-PD1 + XUC group (Figure 4D). These results indicate enhanced immune activation and suggest the effectiveness of XUC monotherapy and combined therapies. However, CD68^+^ monocyte (Figure 4E) infiltration showed no significant differences across all treatment groups, with levels remaining relatively stable: vehicle (87 ± 17.87), XUC (90.25 ± 42.97), GEM (108.8 ± 33.33), GEM + XUC (52.69 ± 42.3), anti-PD1 (75.42 ± 39.12), and anti-PD1 + XUC (67.31 ± 26.98), suggesting that XUC treatment does not substantially impact monocyte recruitment. Notably, CD11b^+^ cells (Figure 4F), which are associated with immunosuppressive activity, were significantly reduced following XUC-involved treatments. In the vehicle control group, CD11b^+^ cell number was measured at 313.8 ± 18.77, whereas XUC monotherapy reduced this to 149.4 ± 29.91. GEM monotherapy showed a minor reduction to 267 ± 19.73. The combination treatments led to further decreases in cell number, with GEM + XUC at 134.9 ± 17.35, anti-PD1 at 162.2 ± 21.89, and the lowest cell number observed in the anti-PD1 + XUC group at 127.6 ± 27.02. These findings suggest that intravesical XUC treatment modulates the immune cell tumor microenvironment, except the CD68+ cell, which showed no significant difference between all treatments and the vehicle control group (Figure 4E) since monocytes could play a dual role in both pro- and antitumoral immunity during tumor progression [24].

### 3.5. Intravesical Xenogeneic Urothelial Cell (XUC) Treatment Enhances Immune Cell Functions

**Figure 5:**
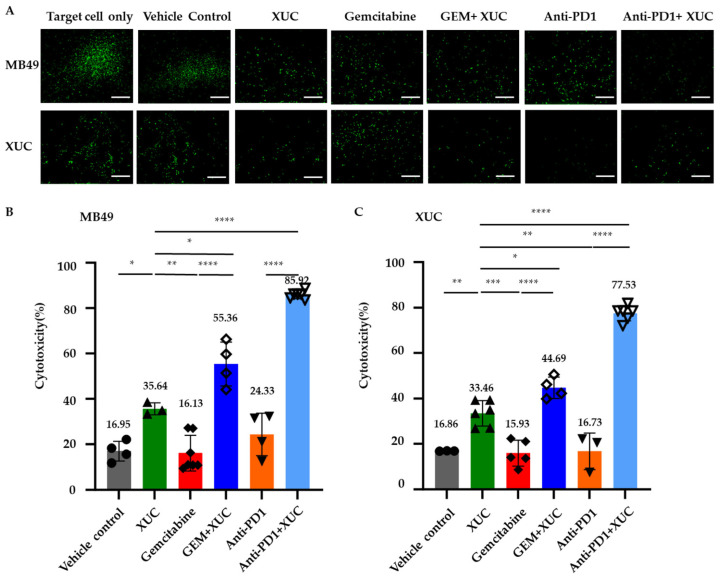
The immune cell cytotoxicity to bladder cancer cells and xenogeneic urothelial cells (XUC) is increased by XUC treatment. After the endpoint of the experiments, immune cells in the spleens of treated mice were harvested to measure cytotoxicity. CFDA-labeled MB49 and XUC were seeded in 96-well plates and co-cultured with immune cells from treated mice at an effector-to-target (E/T) ratio of 10:1. (**A**) Representative fluorescent images of wells showing immune cell-mediated killing of MB49 cells and XUC cells. (**B**,**C**) The relative cytotoxic activity of lymphocytes was quantified using an ELISA reader. The scale bar represents 10 μm. The number on the top of the bar shows the average value. Data are presented as mean ± SD. Statistical significance was determined by one-way ANOVA. * *p* < 0.05; ** *p* < 0.005; *** *p* < 0.005, **** *p* < 0.0001.

Since XUC treatment enhances both TLS formation and tumor-lymphocyte infiltration in the tumors, we next investigated whether the treatments could activate lymphocyte immune functions, including cytotoxicity, proliferation, and cytokine secretion. CD8+ T cell cytotoxicity is a key factor in mediating antitumor effects in cancer immunotherapy [25]. To determine whether XUC immunotherapy enhances T cell cytotoxic function, we conducted an immune effector cell-mediated cytotoxicity assay. Immune cells isolated from the spleens of mice in different treatment groups were used as effector cells. XUC or MB49 cells, labeled with CFDA-SE, served as target cells in the co-culture assay. After 16–18 h of co-culture, immune cells were removed, and CFDA fluorescence intensity was detected (Figure 5A). Cytotoxic activity was calculated based on the formula described in the methods section. The results demonstrated that XUC treatment, either alone or in combination, significantly enhanced immune cell cytotoxicity against MB49 and XUC target cells. The highest cytotoxic activity was observed in the anti-PD1 + XUC combination group, reaching 85.92 ± 1.63% against MB49 target cells and 77.53 ± 3.07% against XUC target cells, significantly surpassing XUC monotherapy (35.64 ± 2.17% and 33.46 ± 5.1%, respectively) and all other treatment groups (Figure 5B,C). In contrast, the vehicle control group exhibited low cytotoxic activity (16.95 ± 3.75% against MB49, 16.86 ± 0.06% against XUC), and GEM monotherapy did not significantly enhance cytotoxicity (16.13 ± 7.24% against MB49 cells and 15.93 ± 5.05% against XUC cells). However, the combination of GEM + XUC resulted in a substantial increase in cytotoxicity (55.36 ± 8.39% against MB49 cells and 44.69 ± 4.06% against XUC cells), demonstrating the added benefit of XUCs. Anti-PD1 monotherapy exhibited moderate cytotoxic activity (24.33 ± 8.12% against MB49 cells and 16.73 ± 6.53% against XUC cells), which was lower than both GEM + XUC and anti-PD1 + XUC treatments. In both cases, the combined treatments induced significantly higher cytotoxicity compared to XUC monotherapy, demonstrating the enhanced immune responses triggered by the combined therapeutic approach.

**Figure 6:**
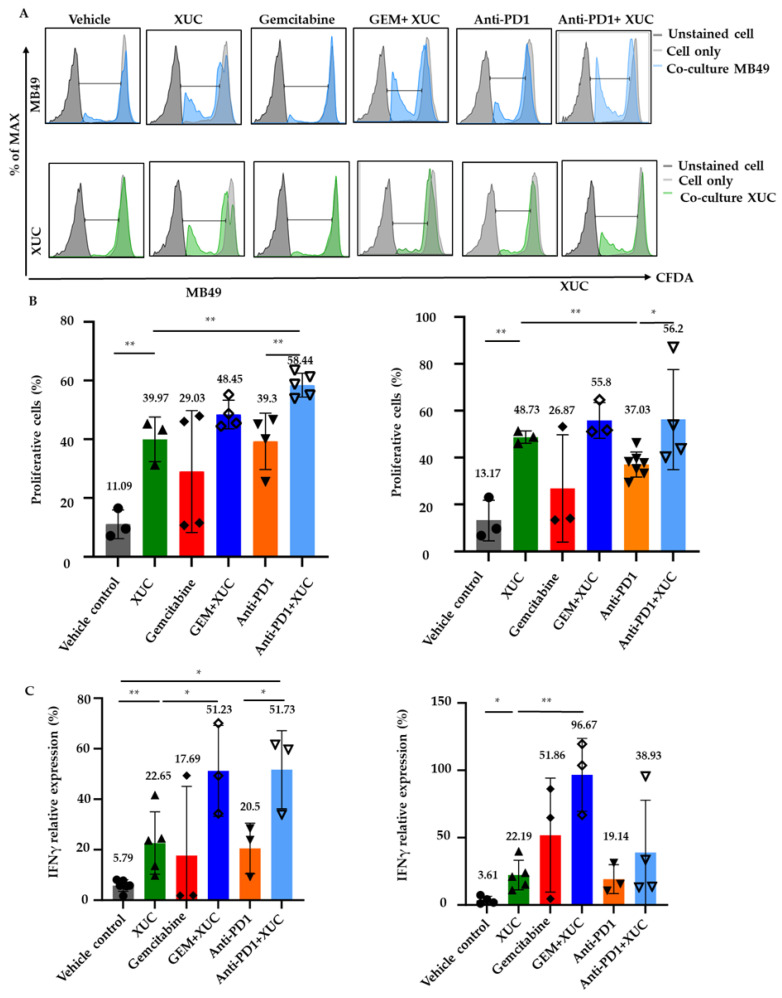
The immune cell proliferation and cytokine secretion assays reveal enhanced immune responses in mice treated with XUC, gemcitabine, and anti-PD1. After co-culturing immune cells from the spleens of treated mice with either bladder tumor cells or for 3 days, CFDA-labeled lymphocytes were collected and analyzed by flow cytometry to determine the proportion of proliferating cells. (**A**) Representative FACS histograms for immune cells are shown. (**B**) The percentage of proliferating immune cells was quantified using FlowJo software (version 10). (**C**) Media from the co-cultures were harvested and analyzed by ELISA to assess the relative expression of IFNγ. The number on the top of the bar shows the average value. Data are presented as mean ± SD. Statistical significance was determined using multiple *t*-tests. * *p* < 0.05, ** *p* < 0.01.

We next performed a co-culture assay to assess whether immune cells from treated mice exhibited enhanced proliferative responses when co-cultured with MB49 cells or XUC. Using CFDA-SE-based proliferation assay, effector immune cells isolated from the spleens of treated mice were labeled with CFDA-SE and co-cultured with either MB49 or XUC target cells for 3 days. Afterward, the immune cells were harvested, and their proliferation capacity was evaluated by flow cytometry, measuring the intensity of CFDA-SE fluorescence intensity in cells. Interestingly, we found that CFDA-SE-labeled effector immune cells stimulated by co-culture with either MB49 cells or XUC showed a higher proportion of proliferating lymphocytes (CFDA-SE low) in mice treated with XUC cells than that of mice treated with vehicle control (Figure 6A). Specifically, approximately a 30% increase in cell proliferation was observed in the co-culture with MB49 cells and a 35% increase in the co-culture with XUC cells (Figure 6B). XUC treatment effectively stimulated effector immune cell proliferation, enhancing immune responses against both target MB49 cells and XUC cells. In co-culture with MB49 cells, XUC monotherapy led to a significant increase in cell proliferation (39.97 ± 6.18%) compared to the vehicle control (11.09 ± 3.95%), while combination treatments further enhanced this effect. Specifically, the GEM + XUC and anti-PD1 + XUC groups exhibited the highest levels of proliferation, reaching 48.45 ± 4.21% and 58.44 ± 3.65%, respectively (Figure 6B). A similar trend was observed in co-culture with target XUC cells, where XUC monotherapy increased cell proliferation to 48.73 ± 2.12%, and the combination treatments with GEM and anti-PD1 further enhanced proliferation to 55.8 ± 6.23% and 56.2 ± 18.48%, respectively, while the vehicle control group showed a proliferative level of 13.17 ± 7.06%. In contrast, GEM and anti-PD1 monotherapies induced moderate effects, with proliferation levels of 29.03 ± 17.94% and 39.30 ± 8.33% against MB49 cells, and 26.87 ± 18.62% and 37.03 ± 4.93% against XUC cells. These findings indicate that XUCs not only enhance immune activation but also synergize with combination therapies to further amplify effector lymphocyte proliferation, reinforcing their role in eliciting a robust antitumor immune response.

We further evaluated the level of IFNγ in co-cultures of effector immune and target cells, which play critical roles in antitumor immunity [26]. The conditioned medium from these co-cultures was collected and assayed for IFNγ activation using the ELISA method. XUC treatment significantly enhanced IFNγ activation in co-cultures of immune cells with both target MB49 tumor cells and XUC cells, indicating a robust immune response. Compared to the vehicle control (5.79 ± 2.09%), XUC monotherapy induced a substantial increase in IFNγ activation (22.65 ± 11.07%) when co-cultured with MB49 cells, while combination therapies with GEM and anti-PD1 further amplified this response to 51.23 ± 14.7% and 51.73 ± 12.63%, respectively (Figure 6C). GEM and anti-PD1 monotherapies also increased IFNγ activation to 17.69 ± 22.4% and 20.5 ± 8.16%, respectively. Similarly, when co-cultured with XUC target cells, IFNγ activation was elevated in the XUC-treated group (22.19 ± 9.87%) compared to the vehicle control (3.61 ± 2.42%), with the GEM + XUC group showing the most pronounced effect (96.67 ± 22.1%). The anti-PD1 + XUC combination also increased IFNγ activation to 38.93 ± 33.72%, while monotherapies of GEM and anti-PD1 resulted in moderate activation (51.86 ± 34.54% and 19.14 ± 8.77%, respectively). These findings suggest that XUC treatment, particularly in combination with GEM or anti-PD1, enhances effector cell cytokine production, correlating with increased lymphocyte proliferation and reinforcing the action of XUC in modulating immune responses in the tumor microenvironment.

### 3.6. Intravesical Treatment with XUC Enhances Immune Effector-Target Cell Formation Between Immune Cells and MB49 Cells

**Figure 7:**
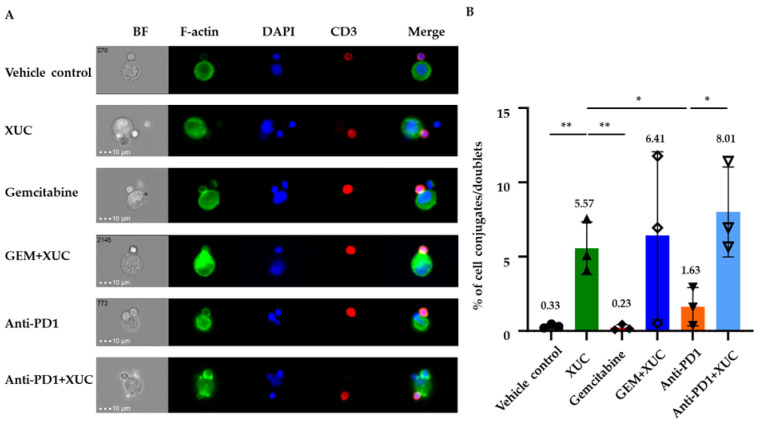
Xenogeneic urothelial cell (XUC) treatment induces the formation of immune effector-target cell conjugates. Isolated immune cells from the spleens of each treated group were suspended and co-cultured with MB49 cells for 45 min. Following fixation and staining, the immune effector-target cell conjugates were analyzed by Amnis ImageStream. (**A**) Representative multispectral imaging flow cytometry images showing CD3+ cell/MB49 conjugates. Conjugates between CD3+ cell and MB49 were stained for CD3 (red) and F-actin (Phalloidin, green), with nuclei stained with DAPI (blue). (**B**) Quantification of immune effector-target cell conjugates between CD3+ cells and MB49 was performed, with data represented as immune effector-target cell conjugates/doublets. The scale bar represents 10 μm. The number on the top of the bar shows the average value. Data are presented as mean ± SD. Statistical significance was assessed by multiple *t*-tests. * *p* < 0.05, ** *p* < 0.01.

To evaluate the direct physical interaction between immune cells and tumor cells, we measured the formation of immune effector-target cell conjugates. As the analysis of mature immune effector-target cell conjugate formation relies on F-actin content in immune cells engaged with MB49 cells, imaging flow cytometry was employed to investigate the formation of immune effector-target cell conjugates between immune cells of different treatment groups and MB49 cells. This technique enables the precise analysis of F-actin content in conjugated cells, offering insights into the cytoskeletal dynamics during immune effector-target cell conjugate formation. Immune cells isolated from the spleens of tumor-bearing mice with different treatments were co-cultured with MB49 cells. Figure 7A presents representative images from the T cell singlet gate and the refined cell couple gate. The polymerization of F-actin, as visualized through phalloidin staining, served as a key indicator of mature immune effector-target cell conjugate formation. T cells demonstrated substantial F-actin accumulation at the interface with MB49 cells, underscoring the cytoskeletal reorganization involved in effector-target cell conjugate formation. An effector-target cell conjugate was considered mature when both CD3 and F-actin were enriched at the contact zone between T cells and MB49 cells. XUC treatment significantly enhanced immune effector-target cell conjugate formation compared to the vehicle control (0.33 ± 0.11%), with 5.57 ± 1.45% of mature effector-target cell conjugates observed. The combination of GEM and XUC further increased this interaction to 6.41 ± 4.61%, while the highest proportion of mature effector-target cell conjugates (8.01 ± 2.47%) was detected in the anti-PD1 + XUC treatment group (Figure 7B). In contrast, monotherapies with GEM and anti-PD1 induced minimal conjugate formation (0.23 ± 0.16% and 1.63 ± 1.05%, respectively). These findings highlight XUC’s ability to enhance T cell-mediated tumor interactions, particularly in combination with immunotherapeutic agents. This observation suggests that XUC treatment actively promotes immune effector-target cell conjugate formation in immune cells. The enhanced effector-target cell conjugate formation observed in this study provides valuable insights into the cellular mechanisms driving immune responses in the xenogeneic cell immunotherapy approach.

## 4. Discussion

Due to its high mutation rate, bladder cancer is considered immunogenic and thus responds well to immunologic treatments [27]. Intravesical instillation of Bacillus Calmette–Guérin (BCG) was the first cancer immunotherapy for NMIBC since its first therapeutic application in 1976 [28], followed by the approval of pembrolizumab, Nadofaragene firadenovec, and nogapendekin alfa inbakicept plus BCG. There are other immune approaches still in development, such as intravesical CG0070, including a replication-competent oncolytic adenovirus that targets bladder tumor cells through their defective retinoblastoma pathway with the expression of granulocyte-monocyte colony-stimulating factor transgene, which has shown promising activity in patients with high-grade non-muscle-invasive bladder cancer (NMIBC) who previously did not respond to bacillus Calmette–Guérin (BCG) [29]. All of these treatments harness some functions of the body’s immune system to treat cancers, but the effects are still limited and come with side effect concerns.

The present preclinical study supports the antitumor efficacy of intravesical xenogeneic urothelial cells in a single treatment using a well-established mouse bladder cancer cell line, MB49 graft non-muscle-invasive bladder cancer (NMIBC) model, and the combined therapies with either chemotherapy or ICI have more profound effects. As a single treatment agent, XUC treatment reduced tumor burden by 60% compared to vehicle control, whereas the combination of XUC with chemotherapy or ICI further repressed tumor development. The cellular and molecular results have further shown an altered tumor immune microenvironment from suppressive to active by stimulating the infiltration of CD4+, CD8+ T-cells, and NK cells, as well as decreasing immunosuppressive cells. Immune function assays for cell proliferation, cytotoxicity, and cytokine secretion, as well as effector/target cell interaction, suggested that the antitumor responses were linked to the increased cell growth, killing capacity, secretion of IFN cytokine, and the effector-target cell conjugation. The combinational treatment had significantly higher immune responses than single XUC treatment.

Based on the results that increased immune cell responses in cytotoxicity, proliferation, and cytokine secretion by the treatment of xenogeneic cells to both xenogeneic cells and tumor cells, we inferred that the intravesical administration of XUC will generate immunologic responses from rejection to antitumor immunity due to the similarities between xenoantigens and tumor-associated and specific antigens, leading to significant bladder tumor progression inhibition. We hypothesized that molecular mimicry or epitope (determinant) spreading could play a role in the antitumor immunity elicited by xenogeneic cells. Molecular mimicry occurs when there is a structural similarity between self and non-self-epitopes in the case of viral infections, which is able to elicit the activation of cross-reactive B and/or T cells, resulting in autoimmunity [30]. Epitope spreading is described as the T-cell-mediated and antibody-mediated immune responses to endogenous epitopes secondary to the release of self-antigens during virus infections and autoimmune disease, when tissue damage caused by inflammation leads to the release of large quantities of different “leaking” autoantigens to then generate and expand a cascade of autoreactive immune cells [31,32]. Epitope spreading has also been linked to the effectiveness of antitumor immune response in cancer immunotherapy [33]. Thus, we suggested that in xenogeneic cell immunotherapy, molecular mimicry (between xenoantigens and neoantigens) or epitope spreading (from xenoantigens to neoantigens) mechanisms elicited by xenoantigens on xenogeneic cells induce tumor-specific T cells and B cells to proliferate and function with the release of cytokines and chemokines as well as antibodies. Working together with cytotoxic immune cells like T cells and NK cells, the activated all-out immune responses could then eliminate malignant cells with every possible immunological reaction. In this scenario, autoimmunity could occur due to molecular mimicry or epitope spreading elicited by xenogeneic cells. However, in the studies of heterotopic pig-to-primate cardiac xenotransplantation, the pig hearts were placed in the non-human primate recipient’s abdomens, xenograft rejection still occurred on genetically modified pig hearts under immunosuppression, but autoimmunity was not reported in the recipient’s native hearts and bodies [34,35,36]. These studies suggest that our xenogeneic cell immunotherapy approach may only induce xenogeneic rejection and antitumor immunity, but not autoimmunity.

Compared to the BCG with xenogeneic urothelial cells, both activate CD4+ T cells, CD8+ T cells, and NK cells as well as the production of cytokines, such as IFN-γ, to mediate their antitumor effects [37,38]. However, BCG, a viable, living organism, can cause infections, resulting in side effects ranging from bothersome cystitis in the majority of patients to sepsis, which could lead to death in rare cases [39]. On the other hand, xenogeneic urothelial cells are isolated from normal porcine urothelium, which was shown to have tissue-repairing ability for chemical-induced cystitis in a murine model [40]. Furthermore, the global BCG shortage has been a healthcare concern that impacts both providers and patients [41]. Thus, the development of new efficacious treatments to replace BCG is essential. Furthermore, intravesical treatment after TURBT is aimed at preventing tumor cell implantation to the injury site by the surgery and treating undetected small cancer lesions in the urothelium. Observations from this study, and previous reports, show that intravesically administered xenogeneic cells are able to adhere to urothelium and trigger local immune responses [40]. Intravesical urothelial cell treatment could be a potential option for BCG replacement in sequence or combined therapies with other therapeutic modalities.

## 5. Conclusions

The single treatment of intravesical xenogeneic urothelial cells activates both innate NK and adaptive T cell-mediated immune responses to inhibit tumor progression and combinations with chemotherapy or ICI have a better effect. This novel therapeutic approach has the potential as a backbone treatment to activate all-out immunity and when in combination regimens with other therapeutic modalities to achieve a comprehensive management approach to preserve the bladder for a long-term durable response. Thus, intravesical xenogeneic urothelial cell immunotherapy is a promising option as a novel bladder-sparing therapy and BCG replacement for NMIBC patients.

## Data Availability

The original contributions presented in this study are included in the article. Further inquiries can be directed to the corresponding author.

## Abbreviations

The following abbreviations are used in this manuscript:

NMIBC	Non-muscle-invasive bladder cancer
BCG	Bacillus Calmette–Guérin
XUC	Xenogeneic urothelial cells
PD-1	Programmed death-ligand 1
GEM	Gemcitabine
IHC	Immunohistochemistry
TUNEL	Terminal deoxynucleotidyl transferase (TdT)-mediated dUTP nick end labeling
TLS	Tertiary lymphoid structure
